# Niche-mediated bacterial community composition in continental glacier alluvial valleys under cold and arid environments

**DOI:** 10.3389/fmicb.2023.1120151

**Published:** 2023-03-09

**Authors:** Xianke Chen, Xiangning Qi, Ge Ren, Ruiying Chang, Xiang Qin, Guohua Liu, Guoqiang Zhuang, Anzhou Ma

**Affiliations:** ^1^Research Center for Eco-Environmental Sciences, Chinese Academy of Sciences, Beijing, China; ^2^Sino-Danish College, University of Chinese Academy of Sciences, Beijing, China; ^3^Sino-Danish Center for Education and Research, Beijing, China; ^4^College of Resources and Environment, University of Chinese Academy of Sciences, Beijing, China; ^5^National Institute of Metrology, Beijing, China; ^6^Institute of Mountain Hazards and Environment, Chinese Academy of Sciences, Chengdu, China; ^7^Qilian Shan Station of Glaciology and Eco-Environment, State Key Laboratory of Cryospheric Science, Northwest Institute of Eco-Environment and Resources, Chinese Academy of Sciences, Lanzhou, China

**Keywords:** core bacteria, structure characteristic, composition pattern, adaptive difference, continental glacier

## Abstract

**Introduction:**

Bacteria are an essential component of glacier-fed ecosystems and play a dominant role in driving elemental cycling in the hydrosphere and pedosphere. However, studies of bacterial community composition mechanisms and their potential ecological functions from the alluvial valley of mountain glaciers are extremely scarce under cold and arid environments.

**Methods:**

Here, we analyzed the effects of major physicochemical parameters related to soil on the bacterial community compositions in an alluvial valley of the Laohugou Glacier No. 12 from the perspective of core, other, and unique taxa and explored their functional composition characteristics.

**Results and discussion:**

The different characteristics of core, other, and unique taxa highlighted the conservation and difference in bacterial community composition. The bacterial community structure of the glacial alluvial valley was mainly affected by the above sea level, soil organic carbon, and water holding capacity. In addition, the most common and active carbon metabolic pathways and their spatial distribution patterns along the glacial alluvial valley were revealed by FAPTOTAX. Collectively, this study provides new insights into the comprehensive assessment of glacier-fed ecosystems in glacial meltwater ceasing or glacier disappearance.

## 1. Introduction

Cryospheric hydrological processes not only significantly affect river runoff downstream but also affect ecosystem changes through the water cycle. The glacial meltwater from mountain glaciers is a major source of recharge for inland river basins and plays an essential role in regulating river runoff, especially for arid and semi-arid regions (Slemmons et al., 2013; Yang et al., 2019; Yao et al., 2022). Moreover, global climate change has accelerated the rate of glacier melting and results in the release of large volumes of water, nutrients, and microorganisms into the downstream ecosystems (Slemmons et al., 2013; Knight and Harrison, 2014; Peter and Sommaruga, 2016; Milner Alexander et al., 2017; Kong et al., 2019; Tolotti et al., 2020). Thus, the melting of glaciers is not only vital for aquatic organisms, agricultural water, domestic water, and other basic freshwater supply but also plays a dominant role in driving the ecological evolution of rivers and other landscapes in the downstream semi-arid and desert regions. The glacier-fed river bacteria are fundamental components of aquatic ecosystems, and they are essential for coupling biogeochemical cycles between the pedosphere and hydrosphere. However, it remains unclear how the biodiversity and habitats of these glacier-fed rivers would change under the scenarios of glacial meltwater ceasing or glacier disappearance, in particular in the mountain glaciers of continental climate (Cauvy-Fraunié and Dangles, 2019; Bourquin et al., 2022; Liu et al., 2022). Therefore, under the condition of glacial meltwater cessation, understanding the structural patterns of microbes in the alluvial valley is a basic clue for thoroughly comprehending the soil microbial ecosystem shaped by the cryosphere hydrology and other environments.

These glacier-fed ecosystems are usually considered to be harsh habitats characterized by low temperature, scarce nutrients, and high loss of substrate or sediment, especially in river systems (Milner Alexander et al., 2017). Microorganisms are the major pioneer colonizers and are extensively involved in the elemental cycles of glacier-fed ecosystems and the different succession stages in the cryosphere (Hotaling et al., 2017; Brighenti et al., 2019; Bourquin et al., 2022). In general, Cyanobacteria, Proteobacteria, Actinobacteria, Bacteroidetes, Acidobacteria, Chloroflexi, Verrucomicrobia, Firmicutes, and Gemmatimonadetes are ubiquitous in aquatic ecosystems of the cryosphere (Wilhelm et al., 2013; Hotaling et al., 2017; Kong et al., 2019; Zhang et al., 2021; Bourquin et al., 2022). Studies in the last several decades have mainly focused on species diversity and microorganism traits in aquatic ecosystems (Hotaling et al., 2017). Recently, systematic studies of microbial communities in glacier-fed aquatic systems were reported, including bacterial community diversity and composition, bacterial ecological networks, keystone species, and succession of microbial communities (Kong et al., 2019; Zhang et al., 2021; Ren and Gao, 2022). In addition to the soil as a microbial habitat, a broad range of edaphic variables, such as pH, moisture, and nutrient content, were recognized to influence microbial community diversity and structure (Fierer, 2017). Along with global climate change, the retreat of terrestrial glaciers and the loss of permafrost may affect the microbial biodiversity of glacier-fed rivers. Most previous studies on the glacier-fed alluvial valley microbiome have focused on the glacial melting period (Zhang et al., 2021). However, there is still insufficient knowledge about the spatial patterns of microbial community composition and its driving mechanisms in cold and arid environments (Bourquin et al., 2022).

Soil microorganisms are major drivers of biogeochemical cycling and play critical roles in ecosystem functions, such as regulating soil carbon dynamics and mediating nutrient cycling (Wagg et al., 2014). According to the bacterial community composition characteristics, the concept of core and unique was commonly utilized for host-associated and free-living microbiomes, such as gut microbiomes, rhizosphere microbiomes, and microbiomes of soil or lake ecosystems (Turnbaugh et al., 2009; Shade and Handelsman, 2012; Shade and Stopnisek, 2019; Mo et al., 2021). Generally, the classical studies of soil microbiota have mainly focused on the core taxa of the microbial communities because the core species (or OTUs) are essential for understanding the ecological evolution, assembly, and composition and function of microbial communities or they are most important and active in the biogeochemical cycling (Shade and Handelsman, 2012). Meanwhile, recent studies have also emphasized the ecological importance of the unique taxa due to the functional dissimilarity of unique members to the core taxa, which could provide unique functions to support the ecological functioning of the overall community (Lynch and Neufeld, 2015). However, from the perspective of core, other, and unique microbiome, the community composition and potential functional changes of bacteria in the glacial alluvial valley and the potential linkages to the glaciers remain unknown.

Here, we surveyed the geographical characteristics and soil physicochemical parameters, investigated the bacterial community structure and diversity using high-throughput 16S rDNA sequencing, and predicted their potential ecological functions in the glacial alluvial valley habitats. By using the core, unique, and other taxa as a basis for studying the bacterial composition, we analyzed the spatial distribution characteristics and patterns of bacteria and their potential ecological functions of the alluvial valley. The bacterial community structure was identified in relation to geographical characteristics and soil physicochemical properties. Finally, the spatial composition patterns of bacteria in the glacial alluvial valley were discussed, which are of great significance for a comprehensive assessment of glacier-fed ecosystems.

## 2. Materials and methods

### 2.1. Study site description and sampling

**Figure 1:**
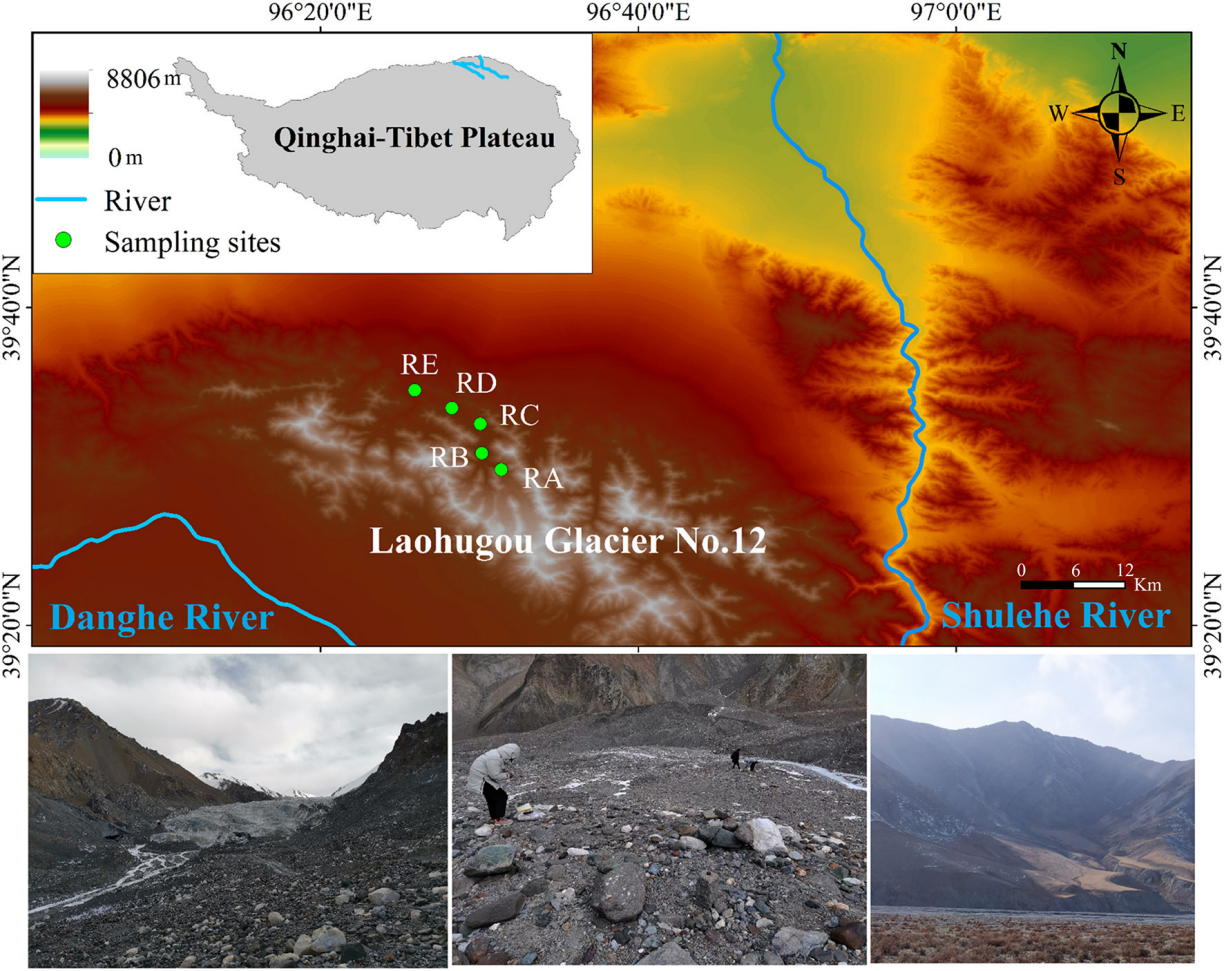
Schematic diagram of the study area. RA, RB, RC, RD, and RE represent five sampling sites in the alluvial valley of the Laohugou Glacier No. 12, respectively. For detailed information on sampling sites, refer to the Materials and methods section.

For this study, Laohugou Glacier No. 12 (a typical composite valley-type glacier, including two major glaciers in the east and west), the largest continental glacier in the Tibetan Plateau, was selected (39°26.4′N, 96°32.5′E, 4,260–5,480 m a.s.l., an area of 20.37 km^2^) (Figure 1). Soil samples were collected in November 2020 in the alluvial valley of the Laohugou Glacier No. 12 and surface soil (-10 cm) samples were obtained in triplicate at each site. Based on the alluvial valley shape and glacial meltwater directional flow of traces, the positions of the five sampling sites retreated from the ice tongue to 0.005 km (39°29′45.13“N, 96°31′25.25”E, 4,278 m a.s.l.), 2 km (39°30′33.18“N, 96°30′19.06”E, 4,155 m a.s.l.), 6.5 km (39°32′44.63“N, 96°30′00.88”E, 4,029 m a.s.l.), 10.5 km (39°34′24.65“N, 96°27′41.08”E, 3,864 m a.s.l.), and 13.5 km (39°34′55.39“N, 96°25′53.41”E, 3,736 m a.s.l.) (denoted RA, RB, RC, RD, and RE, respectively) (Figure 1). After sieving to remove the stones and large particles, soils (< 2 mm) were immediately transported to the laboratory and stored at −80°C until further analysis. Each sample was divided into two parts for the determination of physicochemical properties and DNA extraction, respectively.

### 2.2. Measurements of soil physicochemical factors

The determination of the physical parameters of the soil was performed as described previously (Garaycochea et al., 2020; Wang et al., 2020), including pH, soil water content (SWC), water holding capacity (WHC), total carbon (TC), total nitrogen (TN), and total phosphorus (TP). The potassium dichromate method was used to measure soil organic carbon (SOC) in 3-g air-dried soil samples. Soil-dissolved organic carbon (DOC) was extracted by adding 50 ml of ddH_2_O to 5 g of soil, which was shaken for 24 h at 30°C and filtered through a 0.45-μm filter (Millipore), and determined using an Elementar TOC analyzer (Vario TOC; Elementar). The contents of ammonia-nitrogen (NH${}_{4}^{+}$) and nitrate nitrogen (NO_3−_) in soil were determined using a continuous flow analyzer (AA3; SEAL) (Zhang et al., 2022). Abbreviations: D, geographical distance; a.s.l., above sea level.

### 2.3. Soil DNA extraction, amplification of the 16S rRNA gene, and sequencing

DNA was extracted from 0.5 g of thoroughly mixed soil using a MOBIO PowerSoil DNA Isolation Kit (MOBIO Laboratories, Carlsbad, CA USA) according to the kit manufacturer's instructions. For each sample, soil DNA was extracted in triplicate and pooled into one tube. DNA concentration and purity were measured using a NanoDrop 2000 (Thermo Fisher Scientific, MA, USA).

The extracted DNA was amplified using the primers 341F (5′-ACTCCTACGGGAGGCAGCAG-3′) and 806R (5′-GGACTACHVGGGTWTCTAAT-3′) in the V3-V4 regions of the 16S rRNA gene to characterize the bacterial community (Frey et al., 2016). PCR reactions were performed in a 50 μl reaction system, containing 25 μl of 2 × Premix Taq (TaKaRa), 1 μl of both 10 μM forward and reverse primers, and 3 μl of the DNA (20 ng μL^−1^). The thermal cycle conditions were as follows: 5 min at 94°C for initialization; followed by 30 cycles of 30 s denaturation at 94°C, 30 s annealing at 57°C, and 30 s extension at 72°C; 10 min final elongation at 72°C; and then held at 4°C. PCR products were tested by 1% agarose gel electrophoresis and purified using an E.Z.N.A^®^ Gel Extraction Kit (Omega), and the concentrations were measured using Qubit 3.0 (Thermo Fisher Scientific, Waltham, USA) and stored at −80°C. DNA samples were sent for sequencing on the Illumina Nova6000 platform (Guangdong Magigene Biotechnology Co., Ltd. Guangzhou, China). All sequencing data from this study have been deposited in the NCBI under the accession number PRJNA853101.

### 2.4. Raw data processing

The raw reads of the 16S rRNA gene were analyzed through the Galaxy pipeline (http://mem.rcees.ac.cn:8080/) (Feng et al., 2017). In brief, the reads were assigned to different samples according to their barcodes, allowing for a single mismatch, after which the barcode and primer sequences were trimmed. First, forward and reverse reads of the same sequence were merged using FLASH, followed by quality control, and the unqualified sequences were filtered out (Magoč and Salzberg, 2011). Then, operational taxonomic units (OTUs) were generated from the quality-controlled reads using UPARSE (Edgar, 2013) with a 97% sequence similarity threshold, and total sequences per sample were resampled to the minimum sum of OTUs for further community analysis. Finally, taxonomic annotations for the 16S rRNA gene representative sequences were carried out with the Ribosomal Database Project (RDP) classifier based on the SILVA database with 80% confidence (Wang et al., 2007).

### 2.5. Bioinformatic analysis and statistical analyses

The concept of the core microbe and unique microbe was used to clarify the characteristics among niches, such as microbial community evolution, composition, and ecological function transformation (Turnbaugh et al., 2009; Shade and Handelsman, 2012; Shade and Stopnisek, 2019; Mo et al., 2021). Partitioning microbial communities into core and unique taxa based on the presence/absence of microbial communities has contributed to our understanding of microbial community structures and their potential ecological functions. Thus, we defined core and unique taxa based on previous studies (Shade and Handelsman, 2012; Mo et al., 2021), and a detailed description of the core and unique datasets is presented in Supplementary Excel S1. Specifically, OTUs shared in all samples were defined as core taxa, OTUs presented only in each group were defined as unique taxa, and the remaining OTUs were defined as other taxa. In addition, the potential ecological functions of bacteria alluvial valleys were predicted by Functional Annotation of Prokaryotic Taxa (FAPROTAX v.1.2.4) (Louca et al., 2016). We then manually selected five functional groups to create a functional heatmap with different bacterial taxa. Hierarchical partitioning (HP) and Levins' niche breadth analyses were conducted using R software and the R packages “rdacca.hp” and “spaa” (Jiao et al., 2020; Lai et al., 2022).

Testing the data of each group was normally distributed, and then, the differences in soil physicochemical parameters and α diversities among the five sites were tested using ANOVA and Tukey's *post- hoc* tests. The differences in the bacterial community structure among the sampling sites were visualized through principal coordinate analysis (PCoA) based on Bray–Curtis dissimilarity and were further tested *via* permutation multiple analysis of variance (PERMANOVA). The relationships between bacterial community composition and environmental factors were revealed by the Mantel test analysis based on the Pearson correlation. All the statistical analyses and figures were performed using Biozeron Cloud Platform (http://www.cloud.biomicroclass.com/CloudPlatform), OmicStudio (https://www.omicstudio.cn/tool), and GraphPad Prism 7.0 software.

## 3. Results

### 3.1. Geographical characteristics and soil physicochemical properties

Laohugou Glacier No. 12 is located on the northern slope of the western Qilian Mountains, in the upstream region of the Shulehe River basin, adjacent to the deserts and Gobi (Figure 1), and an important natural reservoir in the arid region of Northwest of China (Chen et al., 2020). This region is characterized by a typical continental climate and affected by the westerlies, precipitation, and melting glaciers mainly concentrated in early summer to early autumn, with an annual average temperature of −11.8°C, and cold and dry in the winter (Zhu et al., 2020). Thus, the special climate conditions of the Laohugou No. 12 glacier and the geographical characteristics of the alluvial valley provide an outstanding model system for understanding the spatial pattern of bacterial community composition under the scenarios of glacial meltwater ceasing or glacier disappearance.

The soil physicochemical parameters of the alluvial valley of the Laohugou Glacier No. 12 exhibited significantly different characteristics, mainly displaying an obvious downward trend of SOC, DOC, and TP from upstream (RA) to downstream (RE) and showing a clear upward trend of WHC and NH${}_{4}^{+}$ (Supplementary Figure S1). Other tested soil parameters, such as pH, SWC, TC, TN, and NO${}_{3}^{-}$, showed a fluctuating upward or downward trend with the directional flow of glacial meltwaters. Furthermore, a.s.l. (D) could be significantly positively (negatively) correlated with SOC and TP in alluvial valley soil. The soil exhibited high levels of TC and WHC with the distance of the increased alluvial valley, whereas the opposite was true for a.s.l. (Supplementary Figure S2). In addition, SOC could remarkably positively affect the DOC, SOC, TN, TP, and SWC in the alluvial valley and exhibited a significant negative correlation with WHC and TN (Supplementary Figure S2). These results indicated that the soil physicochemical characteristics exhibited a strong variation with the alluvial valley of glacial meltwater and might play a role in the ecological evolution of microorganisms, specifically in nutrient content (i.e., TC, SOC, TP, and NH${}_{4}^{+}$) (Zhang et al., 2022).

### 3.2. Bacterial community composition patterns in the alluvial valley

**Figure 2:**
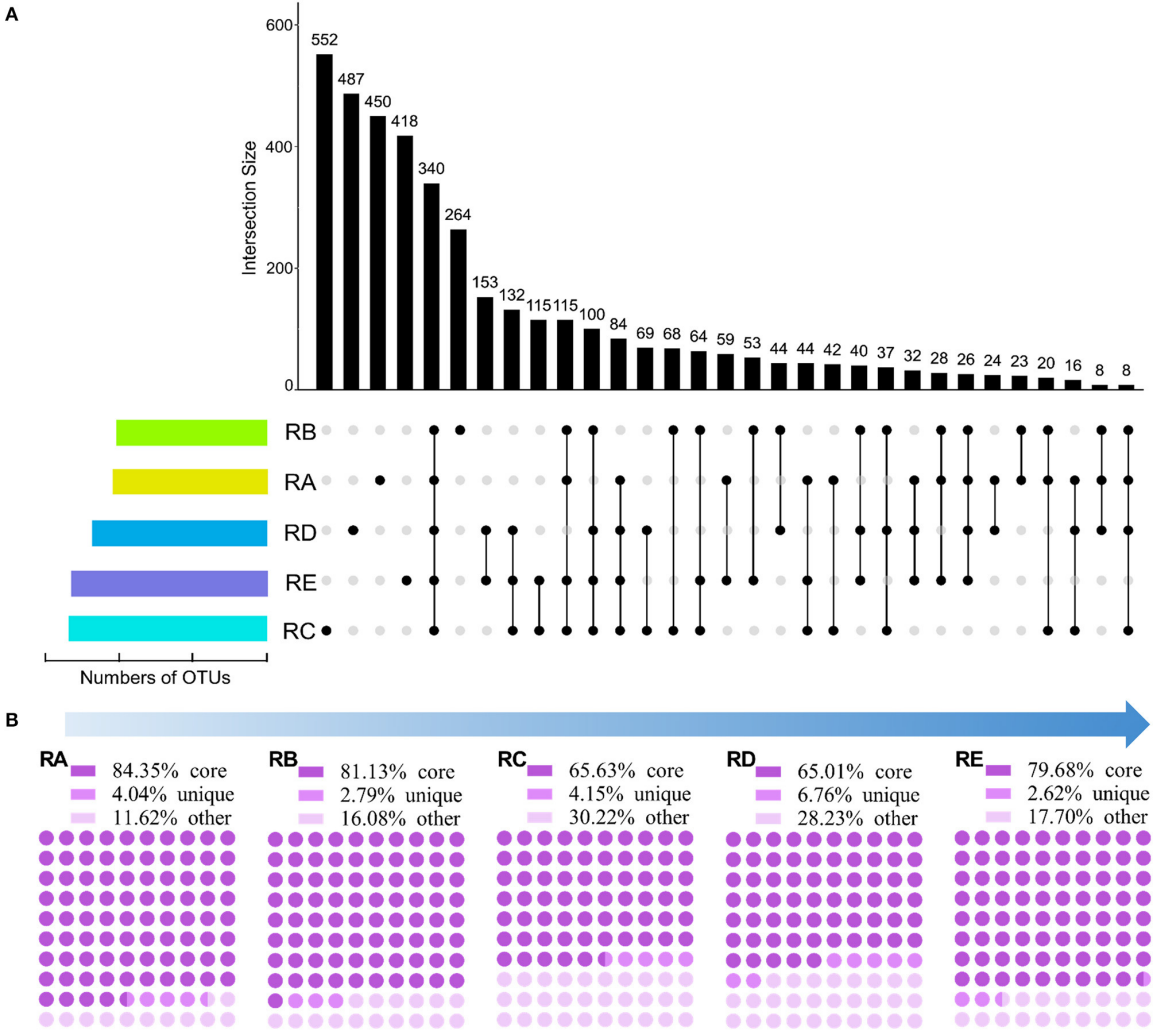
Shared and unique OTUs/sequences in each site. **(A)** UpSet plot showing shared and unique OTUs based on the presence/absence of OTUs with the presence of >70% average OTU abundance mapped alongside. **(B)** The proportion of shared (core), unique, and other sequences based on resample OTU table (rarefied to 36,288 sequences/sample).

In this study, 7,201 OTUs at a 97% sequence identity level were obtained among the 15 soil samples. To explore the specific differences in bacterial community composition patterns among the different geographical environments in the alluvial valley of glacial meltwaters of the Laohugou Glacier No. 12 at the OTU level, an UpSet plot was created to display the OTUs shared and unique taxa and assessed (Figure 2A). The results showed that the numbers of OTUs in RC were the highest (1,806 OTUs), followed by the RE (1,803 OTUs), RD (1,600 OTUs), RA (1,319 OTUs), and RB (1,238 OTUs) (Figure 2A and Supplementary Excel S1). In addition, the numbers of OTUs in both habitats RC (1,806 OTUs) and RE (1,803 OTUs) were similar; however, the proportion of unique taxa in both habitats RC (30.56%) and RE (23.18%) was not comparable (Figure 2A and Supplementary Excel S1). The core taxa showed 25.78, 27.46, 18.83, 21.25, and 18.85% in RA, RB, RC, RD, and RE, respectively (Figure 2A). Furthermore, the shared OTUs in RD&RE were the highest (153 OTUs), followed by the RC&RD&RE (132 OTUs), RC&RE (115 OTUs), and RA&RB&RC&RE (115 OTUs) (Figure 2A). Therefore, these results suggested that mostOTUs in the alluvial valley were core and unique, RA might not recruit more other OTUs, and RC and RE might be the sink.

To further understand the bacterial community structure and composition in the alluvial valley from upstream to downstream, we classified all sequences into three categories based on the UpSet plot and resample OTU table (Figure 2A and Supplementary Excel S1). All samples shared sequences accounted for more than half of the abundance of the total sequences, although shared OTUs accounted for approximately a quarter, with the highest proportions upstream (RA 84.35% and RB 81.13%; Figure 2). The unique sequences in each group accounted for only an extremely small part of the abundance of the total abundance (2.62–6.76%; Figure 2B). The remaining fraction of the total sequences accounted for 11.62–30.22% of the total abundance, namely other sequences that fluctuated greatly (Figure 2B). Moreover, in both habitats RC and RE, the number of core sequences in the RE (79.68%) was greater than the RC (65.63%), although the proportion of shared OTUs in both RC and RE was comparable (Figure 2). Altogether, these findings indicated that core OTU was critical to the bacterial community composition in the alluvial valley. Furthermore, there may be potential to recruit more microbes downstream along the alluvial valley based on core OTU and core sequence.

### 3.3. Diversity and composition of different taxa

The α-diversity of the core and unique bacterial communities (i.e., observed OTUs, Shannon diversity, and Faith's PD) was not significantly different from upstream (RA) to downstream (RE) along the glacial alluvial valley (ANOVA, *p* > 0.05). In addition, the diversity and phylogenetics of all bacterial communities and other bacterial community diversity were not obviously different among the habitats (ANOVA, *p* > 0.05). However, the highest bacterial community richness of all and other taxa was observed in the downstream glacial alluvial valley (Supplementary Figure S3). Furthermore, all bacterial community richness of the RA, RC, and RE sites were not clearly different (ANOVA, *p* > 0.05). PCoA based on Bray–Curtis dissimilarity was performed to investigate the dissimilarity in bacterial community structure, and PERMANOVA was used to determine the difference among the five sites (Supplementary Figure S4). The clear separations of all, core, other, and unique bacterial communities from the five sites were observed along the PCoA1 and PCoA2 axes, which explained 53.15, 65.31, 36.96, and 21.46% of the total variance, respectively, and the dissimilarities of corresponding bacterial communities were all significant (Supplementary Figure S4). This suggests that bacterial community diversity and structure were different at each site and strongly influenced by the geographical characteristics of the alluvial valley.

**Figure 3:**
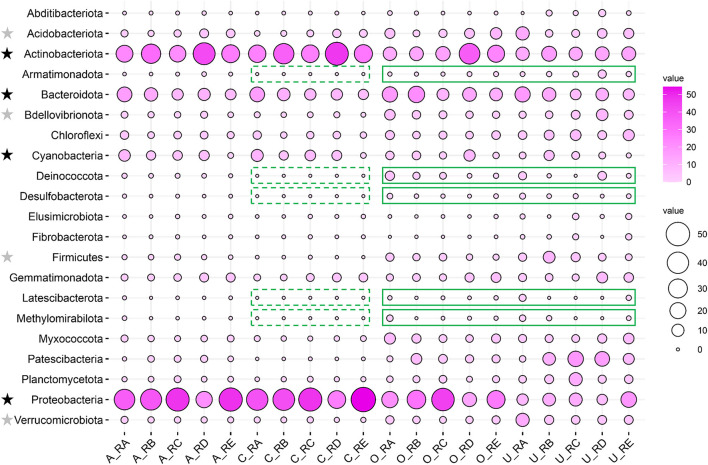
Comparison of bacterial community composition in all, core, other, and unique bacterial taxa in the alluvial valley of the Laohugou Glacier No. 12 glacial meltwaters. The dominant and rare bacteria phyla are marked with a black star and a gray star on the left of the figure, respectively. The core taxa absence and relatively abundant other and unique taxa of the phylum are highlighted by green dotted and solid lines, respectively. The relative abundances of the major phyla (relative abundance >1% at least in each sample) are displayed by a bubble plot.

The relative abundances of the different taxa in the alluvial valley soil samples were investigated at the phylum level. Except for the unclassified, a total of 21 major bacteria phyla (relative abundance of more than 1% at least in one sample) in different taxa were displayed by bubble plot, which together accounted for 92.37–99.83% of the bacterial sequences. The bubble plot for individual sites showed that the distribution of dominant and rare phyla varied across different taxa. The core and all bacterial community compositions were similar in the relative abundance of the phylum level (Figure 3). Furthermore, the dominant bacteria phyla were Proteobacteria (21.00–54.72%), Actinobacteriota (21.50–47.41%), Bacteroidota (6.10–16.38%), and Cyanobacteria (0.84–9.49%), which accounted for more than 80% of the relative abundance in core and all bacterial communities. There were some rare phyla in core and all bacterial communities, such as Acidobacteriota (0.78–4.14% vs. 1.75–12.50%), Verrucomicrobiota (0.51–1.95% vs. 1.69–11.85%), Bdellovibrionota (0.42–1.87% vs. 1.92–8.85%), and Firmicutes (0.005–1.11% vs. 1.08–8.85%) but relatively dominant phyla in other and unique bacterial communities (Figure 3). In particular, Armatimonadota, Deinococcota, Desulfobacterota, Latescibacterota, and Methylomirabilota were absent for the core taxa but were relatively abundant in other and unique taxa (Figure 3).

### 3.4. Potential ecological functions of bacteria in the alluvial valley

**Figure 4:**
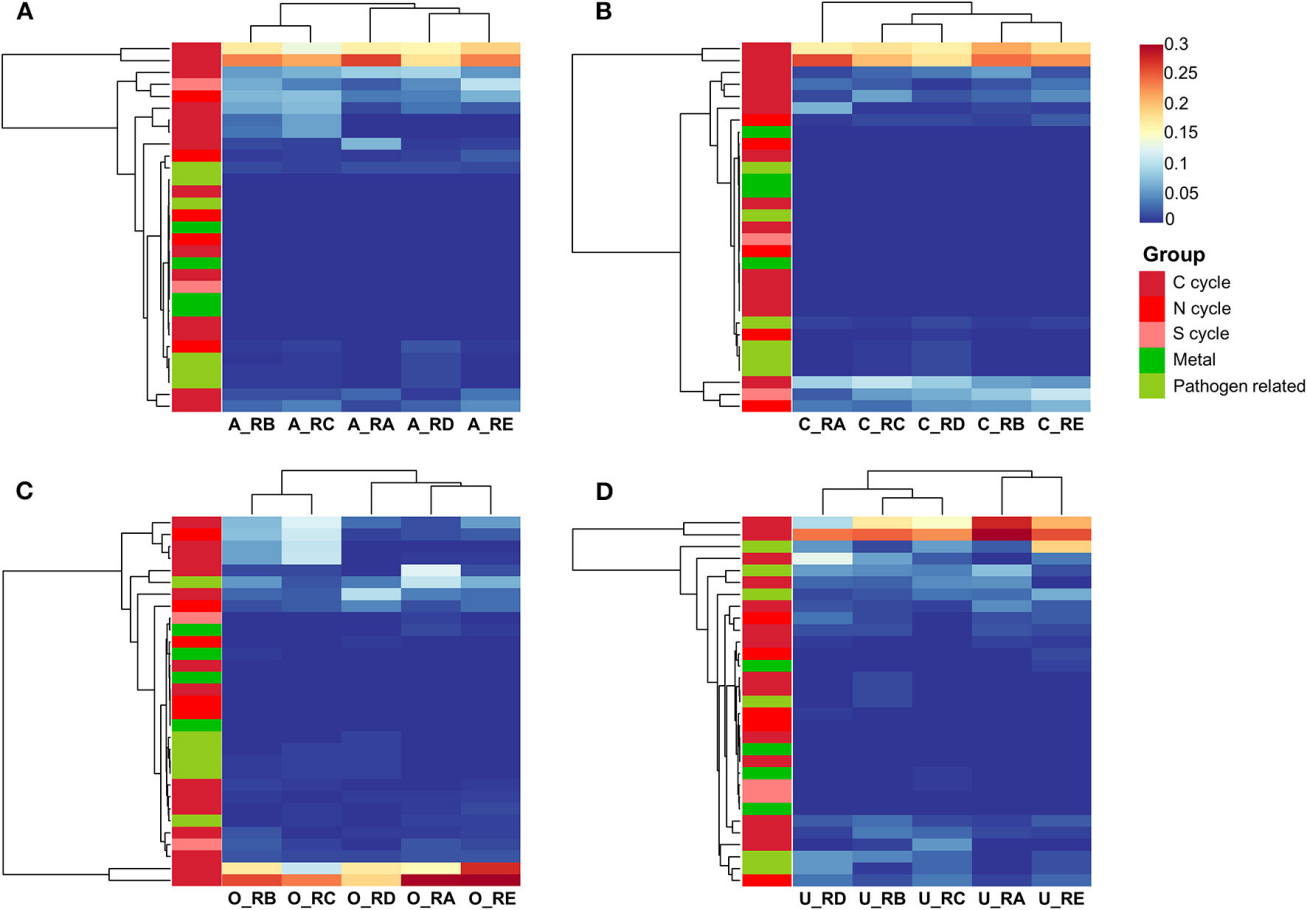
The ecological functions of bacterial communities were explored using FAPROTAX, and the predicted functions were analyzed by a two-way cluster heatmap. All bacteria **(A)**, core bacteria **(B)**, other bacteria **(C)**, and unique bacteria **(D)**. The five colors represent the five functional groups.

Some insights into the bacterial involvement in the biogeochemical cycle of elements in the alluvial valley of Laohugou Glacier No. 12 could be derived from the FAPTOTAX predictions to analyze the potential ecological functions of bacterial communities (Louca et al., 2016). A total of 68, 33, 55, and 57 bacterial functional groups corresponding to 16.64, 32.06, 16.33, and 14.88% of OTUs were identified from the all taxa, core taxa, other taxa, and unique taxa, respectively. Within the identified OTUs, we classified major potential functions into five categories based on the FAPTOTAX prediction results (Figure 4). The functions of most core taxa were focused on the C cycle (except aromatic hydrocarbon degradation), N cycle (except for denitrification), S cycle (except for respiration of sulfur compounds), metal metabolisms (not including iron metabolisms), and degree of pathogen-relatedness (Figure 4B). In addition, except for the unpredictable potential ecological functions of the core taxa, some functions were mainly found in the other taxa, such as denitrification, iron metabolisms, and aromatic hydrocarbon degradation (Figure 4C), whereas the unique taxa exhibited relatively more C cycle and pathogen-relatedness (Figure 4D). In addition, we found a high proportion assigned to the C cycle across all the samples, mainly including aerobic chemoheterotrophy, phototrophy, and chemoheterotrophy (Figure 4). The FAPTOTAX predictions showed that the C cycle was the most common and active metabolic pathway in all identified different bacterial taxa communities. Furthermore, other taxa and unique taxa communities may offer complementary or unique functions to support the ecological functions of the overall bacterial community.

### 3.5. Environmental responses of different bacterial taxonomic compositions

**Figure 5:**
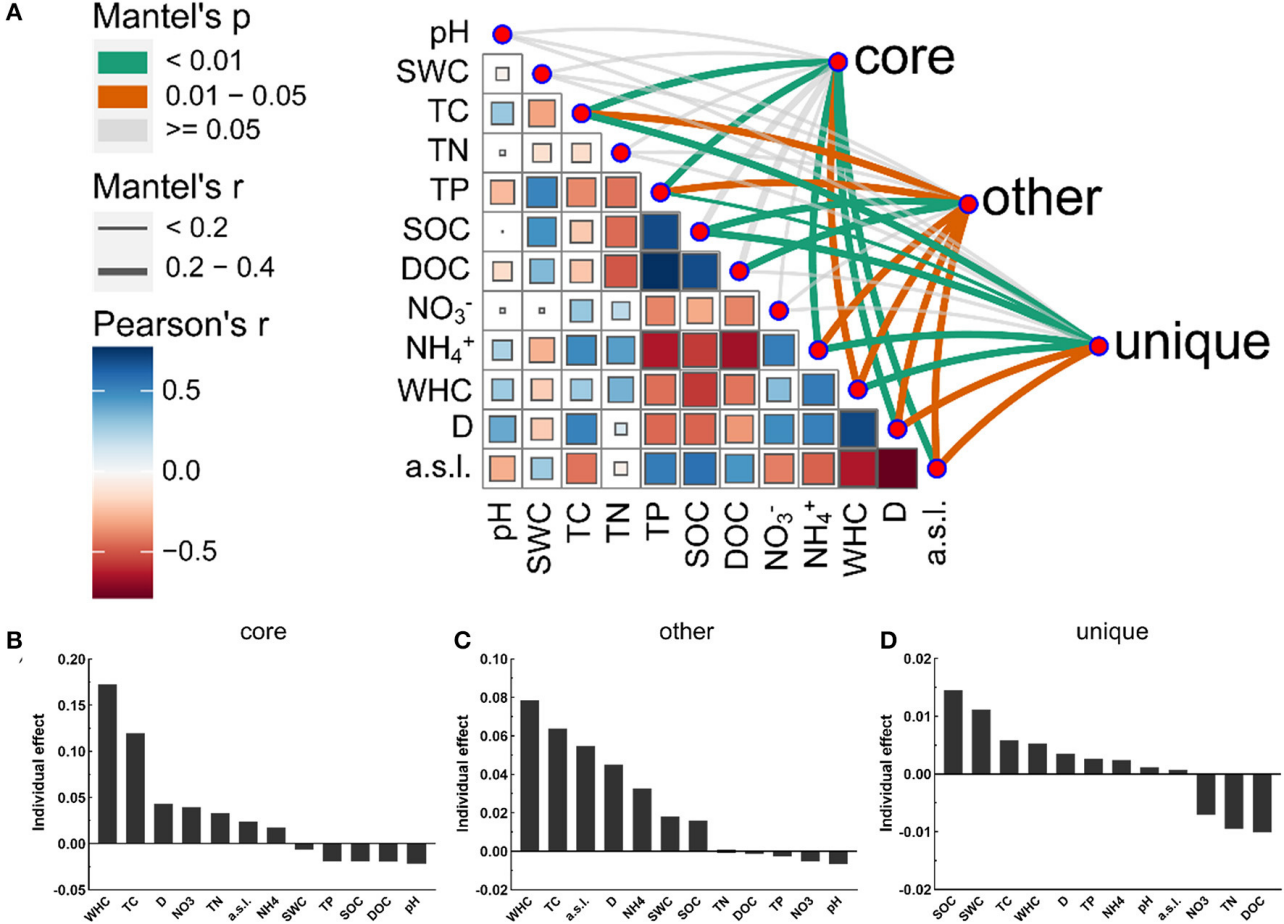
Abiotic drivers of core, other, and unique bacterial community compositions**. (A)** Different bacterial taxa community compositions were correlated with soil physicochemical factors based on the Mantel tests. The line width corresponds to the Mantel *r*-value and the line color indicates the statistical significance based on 999 permutations. Pairwise correlations and Pearson's correlation coefficients of these variables are displayed with circle size and color gradient, respectively. The relative importance of individual environmental variables in the core **(B)**, other **(C)**, and unique **(D)** bacterial community compositions. See Section 2 for definitions of the abbreviations.

A variety of soil physicochemical parameters showed significant correlations with the bacterial community composition of the different taxa. The Mantel tests revealed that the bacterial community composition in the alluvial valley showed significant correlations with geographical characteristics (D and a.s.l.) and soil physicochemical properties (WHC, TC, TP, and NH${}_{4}^{+}$) (Supplementary Figure S5A). The core and all bacterial community compositions were similar in response to soil environmental factors (Figure 5A and Supplementary Figure S5A). In addition, the importance of individual environmental factors was estimated by hierarchical partitioning (HP). HP and Mantel's tests showed similar results, especially for all and core bacterial taxa (Figure 5 and Supplementary Figure S5). Furthermore, the Mantel tests and HP identified SOC as the main variables contributing to the unique taxa of each niche (Figures 5A, D). Taken together, our results demonstrated that the soil physicochemical properties and geographical characteristics modulate different bacterial taxa community compositions. Thus, it is suggested that the bacterial community composition was mediated by the niche.

## 4. Discussion

Climate change is affecting terrestrial and marine ecosystems as we enter the Anthropocene, especially the glacial ecosystem (Webster et al., 2018). For instance, these increasing environmental pressures are forcing the cold adaptation capacity of glacial organisms to reduce and potentially risk local extinction (Wilhelm et al., 2013; Cauvy-Fraunié and Dangles, 2019). In fact, glacier microbiota are essential for biogeochemical cycles in the cryosphere. To date, studies on the cryospheric ecosystems microbiome of mountain glaciers have been relatively scarce, especially in the cold, dry, and glacial non-melting periods (Bourquin et al., 2022). In order to evaluate the fate of microbiota in glacier systems, it is necessary not only to characterize the bacterial community structure within the alluvial valley but also to reveal the factors that drove the composition of bacterial taxa. In this study, we surveyed soil bacteria in the alluvial valley of the Laohugou Glacier No. 12, during the cold and glacial non-melting period (Figure 1). Exploring the spatial microbiome differences and similarities among the different habitats in the alluvial valley deepened our understanding of the ecological composition and functional potential of the cryospheric microbiome and provided general insights into microbiota linking upstream and downstream in the alluvial valley.

Our core and unique taxa composition results showed that the core OTUs and sequences in RA, RB, RC, RD, and RE habitats of the alluvial valley were 25.78 and 84.35%, 27.46 and 81.13%, 18.83 and 65.63%, 21.25 and 65.01%, and 18.85 and 79.68%, respectively (Figure 2). In other words, our results demonstrated that the bacterial core OTU accounts for a relatively low proportion, but they represented the most dominant and abundant bacteria and might constitute the bulk of bacterial community function in the glacial alluvial valley. In addition, bacteria of unique and other taxa constituted a minority of bacterial communities and could be involved in the maintenance of the overall bacterial community function (Figure 4; Lynch and Neufeld, 2015). In addition, this study also observed that the bacterial richness along the alluvial valley showed an overall increasing trend from upstream to downstream, especially for other taxa, but there were no clear trends for other α-diversity (Supplementary Figure S3). As with the all and core bacterial taxa abundance at the phylum level, there was no significant change from upstream to downstream (Figure 3). Together, these results prove the conservative composition and adaptive differences of the cryospheric bacterial community, indicating that the bacterial community composition is dominated by the core taxa, and the unique taxa might fill gaps in the overall bacterial community composition and function in different niches.

Bacterial phylum-level and their underlying ecological function compositions suggested that the bacterial communities in different habitats of the alluvial valley differ both in taxonomical and potential metabolic characteristics. Bacterial composition suggested that the dominant bacteria of the five habitats in the glacial alluvial valley were similar at the phylum level; however, some shared taxa exhibited significantly different compositions and variation patterns, especially Actinobacteriota, Proteobacteria, and Cyanobacteria (Figure 3). Furthermore, some relatively predominant bacteria, such as Desulfobacterota, Latescibacterota, Armatimonadota, Methylomirabilota, and Verrucomicrobiota (Tahon et al., 2018; Choe et al., 2021; Ortiz et al., 2021; Ward et al., 2021; Song et al., 2022), had the capacity to use multiple carbon sources and participate in nitrogen and sulfur metabolism in other and unique taxa, which were consistent with our functional prediction results (Figure 4), implying that other and unique taxa play critical but distinct roles in maintaining whole ecological function and biogeochemical cycles. In addition, it is mainly exhibited that the most important bacterial ecological functions in the alluvial valley were chemoheterotrophy and phototrophy (Figure 4). These heterotrophic and photoautotrophic bacteria, such as Proteobacteria and Cyanobacteria, have key positions in the degradation and production of organic matter in oligotrophic environments of the glacial alluvial valley, respectively (Figures 3, 4). Organic matter produced by cyanobacteria and algae was released into the meltwater and may be carried downstream from upstream of primary production as the ice melts, suggesting that these microbes could strongly affect food webs in glacial ecosystems. This is mainly because environmental selection plays a critical role in shaping microbial community structure, especially glacial microorganisms (Bourquin et al., 2022). Environmental selection could affect the whole microbial community, including core and unique taxa. Moreover, interspecific interactions are also important factors, influencing other microorganisms through mutualism or competitive exclusion, such as some bacteria obtaining fresh C from the primary production of Cyanobacteria (Kong et al., 2019).

Importantly, our findings also indicated significantly different patterns of bacterial community composition and changes across sites, rather than a simple increasing or decreasing trend (Figures 2, 3 and Supplementary Figure S3). Here, we particularly emphasized the anomalies of sites RA, RC, and RE in bacterial community composition and diversity. Compared with sites RB and RD, although site RE has a higher bacterial richness, site RA and RC also seem to have a higher bacterial richness trend (Supplementary Figure S3). This could be elicited in the following ways. First, for site RE, it is well known that the confluence of tributaries, substrates (environmental media), and nutrients from river flow together supported higher microbial diversity during the melting period of glaciers (Brighenti et al., 2019; Kong et al., 2019; Li et al., 2021). Moreover, the better climate downstream of the alluvial valley might be suitable for microbial reproduction and plant growth (Figure 1 and Supplementary Figure S6), which could provide relatively more nutrients for microorganisms. Second, this might be mainly due to the impact of the dam effect on microbial richness at site RC. The dams have significantly altered the upstream and downstream river aquatic ecology and the local environment. As found in previous studies, the upstream of the dam could prolong the retention time of the river, resulting in the increase and accumulation of nutrients, such as organic matter (Liu et al., 2018). According to the more individual hypothesis (Furness et al., 2021), the higher resource availability leads to an increased number of individuals (Supplementary Figure S6), thus supporting higher microbial richness. In contrast, serious sediment erosion and nutrient loss made the downstream of the dam unfavorable for microbial habitat, possibly resulting in lower bacterial abundance (Supplementary Figure S3). Finally, the highest concentration of SOC and the most easily utilizable DOC by bacteria in soil were observed at site RA (Supplementary Figure S1). The bacterial phylum-level and their potential eco-function composition analyses further suggested that a large number of Cyanobacteria would produce large amounts of fresh C through photosynthesis in site RA (Rigonato et al., 2012; Figures 3, 4), which will cause extensive changes to Cyanobacteria-fed biota (Wilhelm et al., 2013; Kong et al., 2019; Supplementary Figure S6), thus supporting higher bacterial richness of the habitat. Taken together, these findings and the potential reasons for these results suggested that niche differences caused by spatial differential, man-made, and biological factors may influence the bacterial community composition of the glacial alluvial valley under cold and arid environments.

Here, our study showed that TC, TP, and SOC were the important driving force shaping bacterial community dynamics and had significant effects, which may result in significant changes in bacterial community composition (Figure 5 and Supplementary Figure S5). In addition, the soil physicochemical parameters showed that TP significantly positively correlated with SOC and DOC in the alluvial valley (Supplementary Figure S2A). This was possibly attributed to the soil P limitation effect, which is widespread in the succession of glacier foreland (Tscherko et al., 2003; Du et al., 2020). To survive, microorganisms stimulate soil organic matter decomposition and obtain relatively more P to support their metabolism by increasing the microbial metabolic quotient (qCO_2_), which may increase CO_2_ release (Spohn and Chodak, 2015; Feng et al., 2021). Specifically, under the condition of soil P limitation, microorganisms can secrete a large number of enzymes (acid phosphatase and alkaline phosphatase) to acquire P from soil organic matter and release its excess carbon, resulting in the increase of qCO_2_ (Spohn and Chodak, 2015; Cui et al., 2020). Meanwhile, a recent study found that soil carbon storage may be reduced because microorganisms used more carbon for enzyme synthesis and P acquisition under the limitation of soil P (Cui et al., 2020). In addition, recent studies indicated that the soil microbe P limitation could reduce terrestrial carbon sinks and might even be transformed into carbon sources (Exbrayat et al., 2013; Du et al., 2020), which may form a positive feedback to the microorganisms in the glacial alluvial valley under climate change conditions. Moreover, our data demonstrated that the WHC significantly modulated bacterial community composition in glacial alluvial valleys under cold and arid environments, suggesting that soil hydrological properties are more important for bacterial community composition in arid and semi-arid regions.

This is a comprehensive study reporting the bacterial community in the alluvial valley of mountain glaciers during the cold, extremely dry, and glacial non-melting period. According to the environmental niche, the diverse bacterial community structures in the alluvial valley of Laohugou No. 12 glacier meltwater show obvious characteristics of changing with the alluvial valley, especially in front of the ice tongue, upstream and downstream of the dam, and downstream of the alluvial valley (Figures 2, 3 and Supplementary Figure S3). Our findings showed that the composition and diversity maintenance mechanisms of the bacterial community structure in the different habitats of the glacial alluvial valley are distinct (Figure 5 and Supplementary Figure S5), which promotes our understanding of microbial ecology in the cryosphere. Meanwhile, the biodiversity of glacier-fed ecosystems will change substantially with glacial meltwater ceasing or glacier disappearance due to climate change in the near future. Therefore, under cold and arid environments, a comprehensive understanding of the spatial composition pattern and its main driving factors of the bacterial community in glacial alluvial valleys is helpful for the study of the succession of bacterial community composition and ecosystem function prediction under climate change.

## Data availability statement

The datasets presented in this study can be found in online repositories. The names of the repository/repositories and accession number(s) can be found in the article/Supplementary material.

## Author contributions

XC, AM, and GZ designed the study. XC and XQi analyzed the data. XC wrote the original manuscript. XQi and GR wrote sections of the manuscript. RC, XQin, and GL provided field data and the database. All authors contributed to the final manuscript.
