# Supplementary material for: Niche-mediated bacterial community composition in continental glacier alluvial valleys under cold and arid environments
Source: Front Microbiol. 2023 Mar 9;14:1120151. doi: 10.3389/fmicb.2023.1120151 (PMC10033870; doi:10.3389/fmicb.2023.1120151)
Supplement: Supplementary file 1 [file Data_Sheet_1.docx]

**Supplementary Material**

**Niche-mediated bacterial community composition in continental glacier alluvial valleys under cold and arid environments**

Xianke Chen^a,b,c^, Xiangning Qi^a,d^, Ge Ren^e^, Ruiying Chang^f^, Xiang Qin^g^, Guohua Liu^a,d^, Guoqiang Zhuang^a,d^, Anzhou Ma^a,d*^

^a^ Research Center for Eco-Environmental Sciences, Chinese Academy of Sciences, Beijing, 100085, China

^b^ Sino-Danish College of University of Chinese Academy of Sciences, Beijing, 101400, China

^c^ Sino-Danish Center for Education and Research, Beijing, 101400, China

^d^ College of Resources and Environment, University of Chinese Academy of Sciences, Beijing, 100049, China

^e^ National Institute of Metrology, Beijing, 100029, China

^f^ Institute of Mountain Hazards and Environment, Chinese Academy of Sciences, Chengdu, 610041, China

^g^ Qilian Shan Station of Glaciology and Eco-environment, State Key Laboratory of Cryospheric Science, Northwest Institute of Eco-environment and Resources, Chinese Academy of Sciences, Lanzhou, 730000, China

*Corresponding author:

Anzhou Ma

Email: azma@rcees.ac.cn

Phone: 86-010-6284-9156


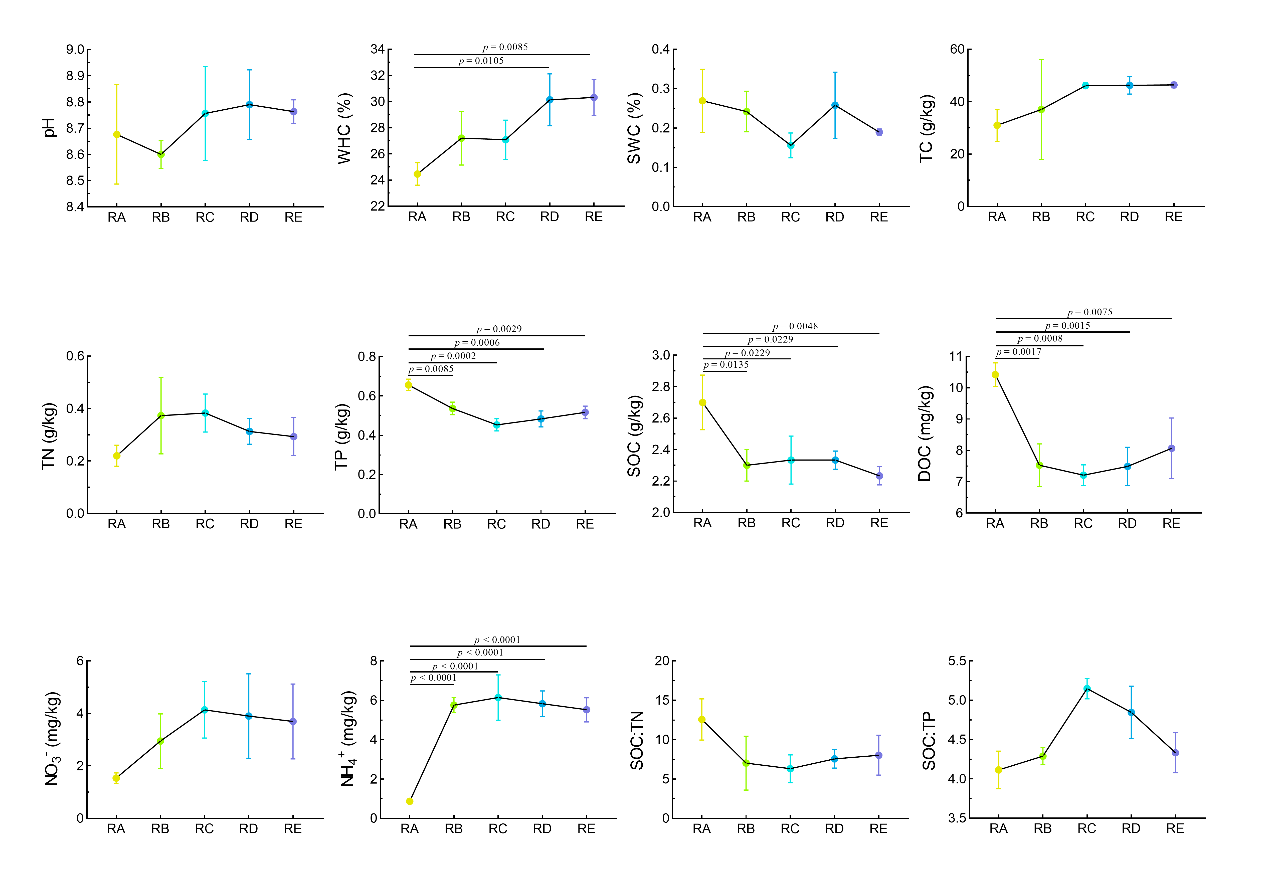


**Fig. S1 Soil physicochemical properties in alluvial valley of the Laohugou Glacier No. 12 glacial meltwaters**. Abbreviations: WHC, water holding capacity; SWC, soil water content; TC, total carbon; TN, total nitrogen; TP, total phosphorus; SOC, soil organic carbon; DOC, dissolved organic carbon; SOC:N, soil ratio of SOC and N; SOC:P, soil ratio of SOC and P.


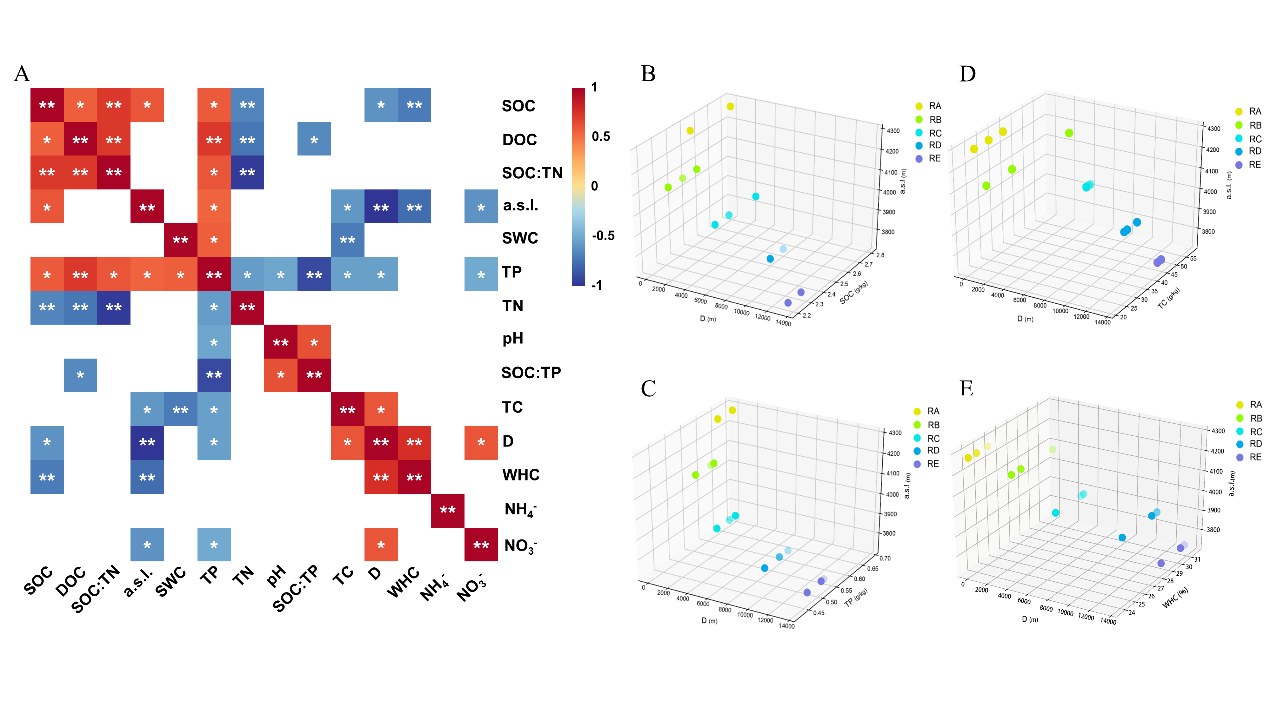


**Fig. S2 Correlation heatmap and 3D schematic among environmental factors.** Heatmap of spearman correlation coefficients analysis among environmental factors (A). The boxes showed in the figure are all significant correlations (P < 0.05 and P < 0.01). Red indicate positive correlations, blue indicate negative correlations, and intensity of the colors indicate the strength of the correlations. The 3D schematic representation of SOC (B), TP (C), TC (D), and WHC (E).


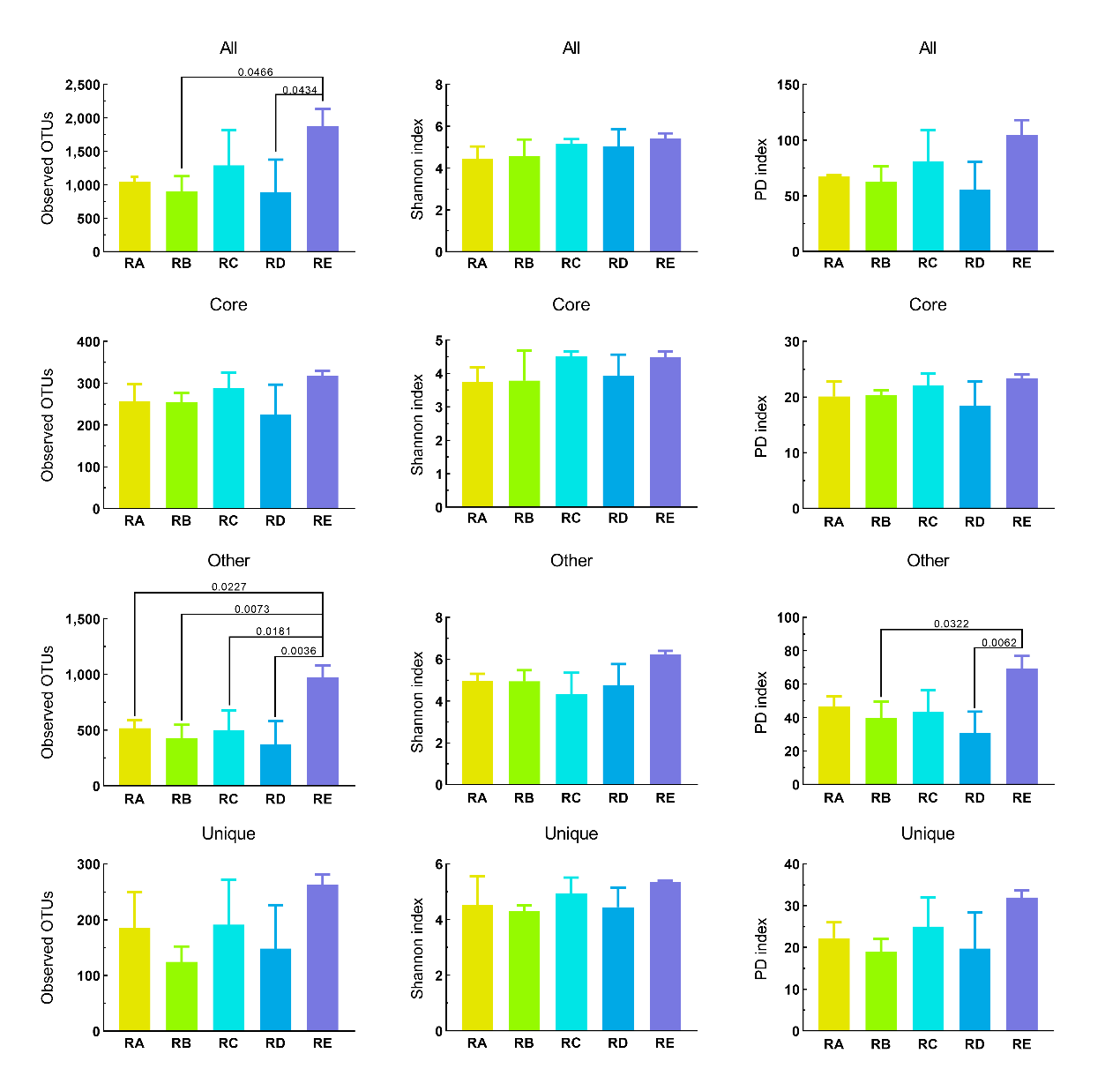


**Fig. S3 Bacterial α diversities at five sampling sites.** Variations in diversity (Shannon index), richness (Observed OTUs), and phylogenetic diversity (PD index) among all OTU, core OTU, other OTU, and unique OTU.


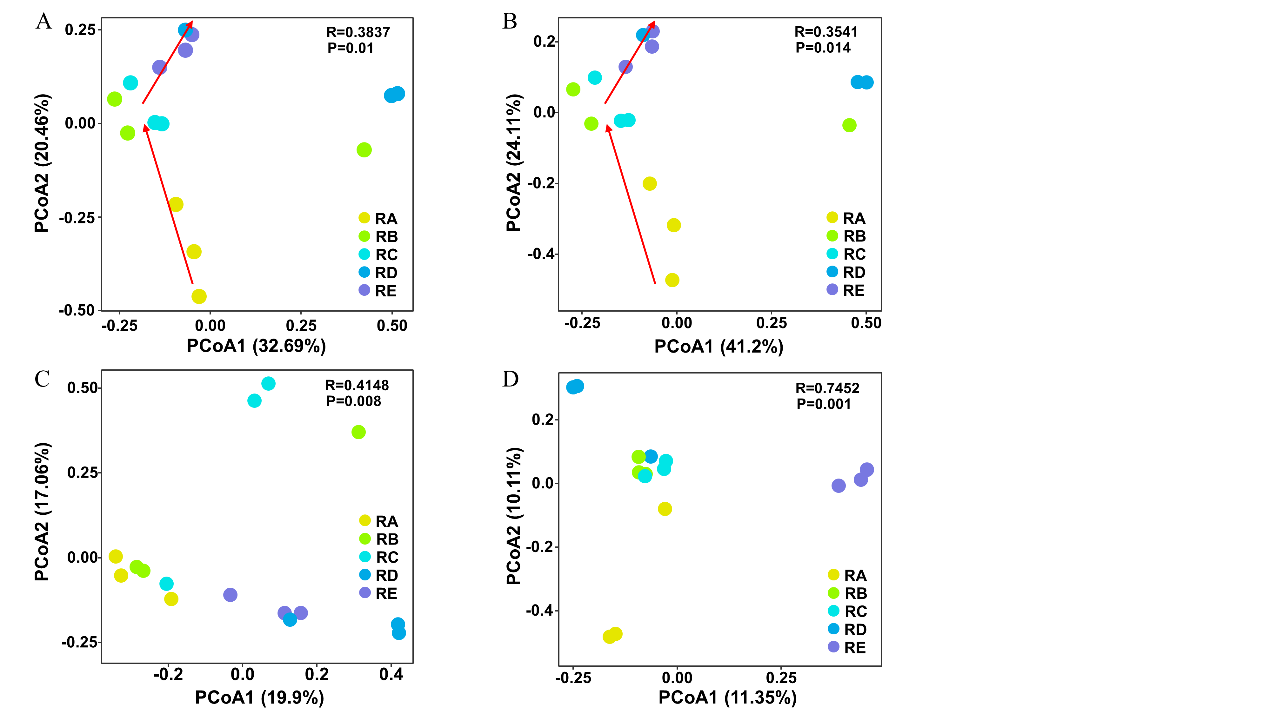


**Fig. S4 Differences in β diversity among the alluvial valley bacteria visualized by principal coordinate analysis (PCoA) based on Bray-Curtis distance.** The all bacteria (A), core bacteria (B), other bacteria (C), and unique bacteria (D). The five colors represent the five sites of sampling.


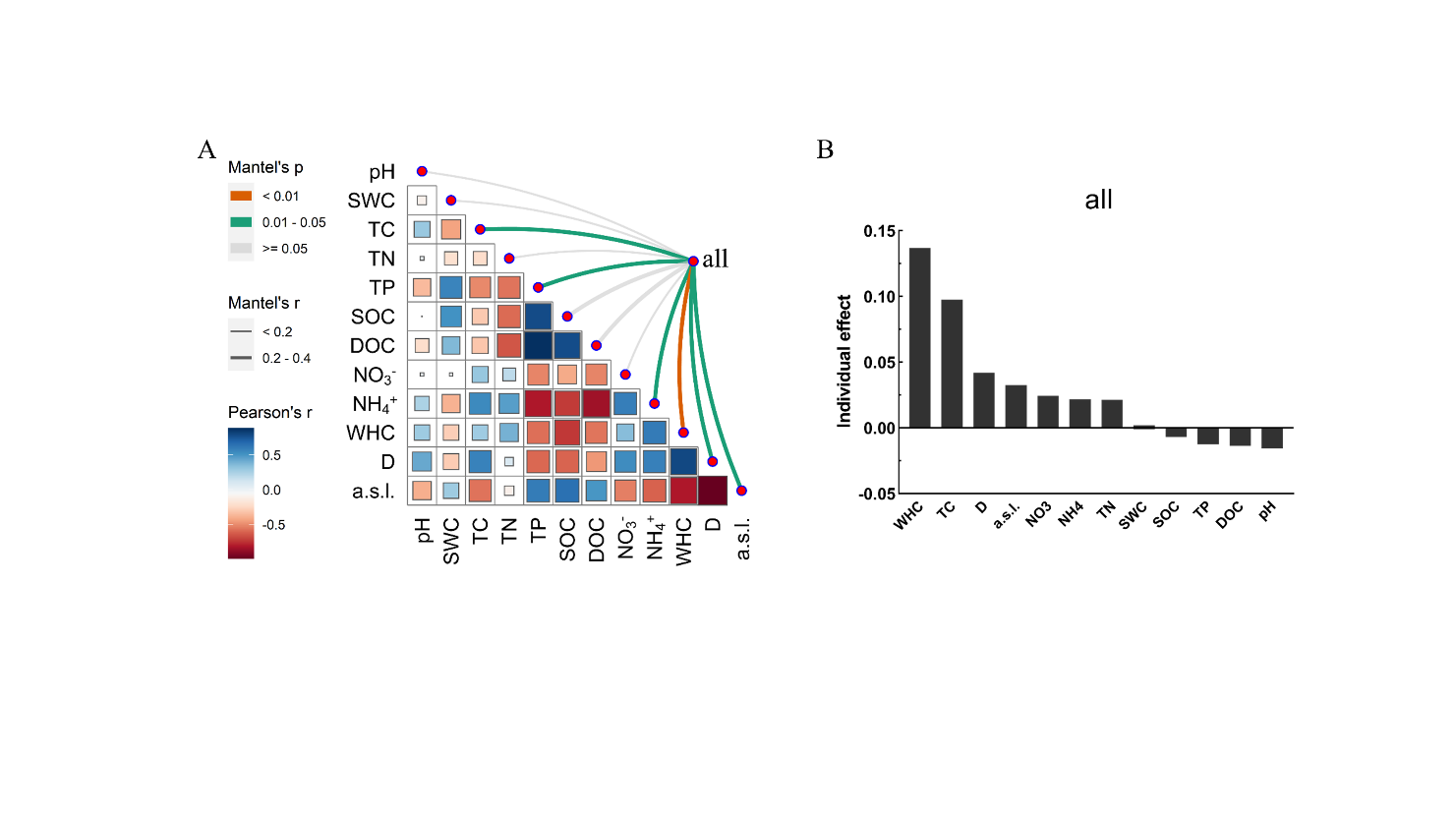


**Fig. S5 Abiotic drivers of bacterial community composition.** (A) The bacterial community composition was correlated to soil environmental factors based on Mantel tests. The line width corresponds to the Mantel’s r value, and the line color indicates the statistical significance based on 999 permutations. Pairwise correlations and Pearson's correlation coefficients of these variables are displayed with circle size and color gradient, respectively. (B) The relative importance of individual environmental variables in the bacterial community composition.


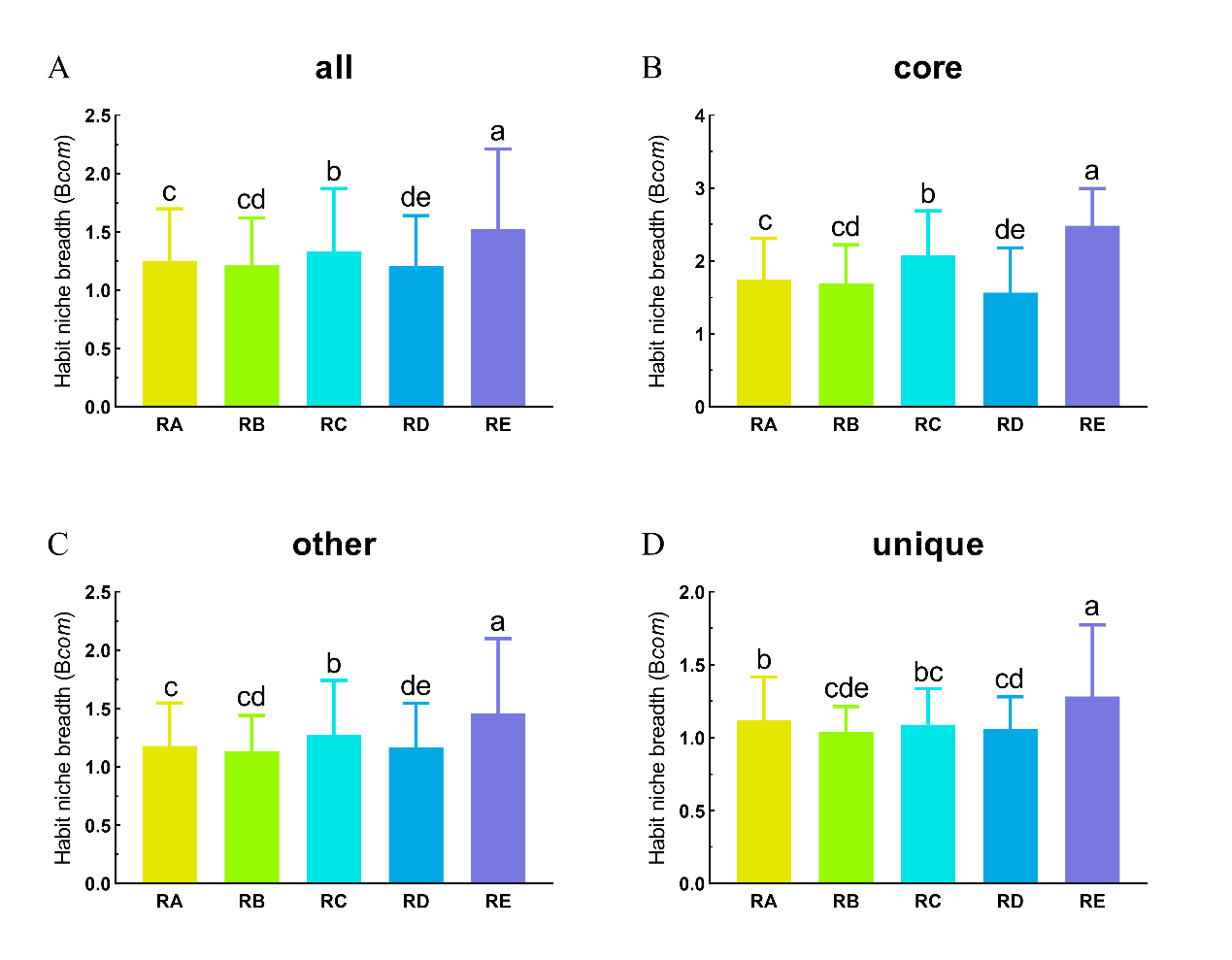


**Fig. S6 Boxplots summarizing the mean niche breadth (B*com*) for different taxonomic bacterial community in RA, RB, RC, RD, and RE sites of the alluvial valley.** The all bacteria (A), core bacteria (B), other bacteria (C), and unique bacteria (D).The significance was assessed by calculating a Kruskal-Wallis test.
